# Motor-Sensory Recalibration Modulates Perceived Simultaneity of Cross-Modal Events at Different Distances

**DOI:** 10.3389/fpsyg.2013.00046

**Published:** 2013-02-26

**Authors:** Brent D. Parsons, Scott D. Novich, David M. Eagleman

**Affiliations:** ^1^Department of Neuroscience, Baylor College of MedicineHouston, TX, USA; ^2^Department of Psychiatry, Baylor College of MedicineHouston, TX, USA

**Keywords:** time perception, motor-sensory recalibration, intentional binding, simultaneity, temporal order

## Abstract

A popular model for the representation of time in the brain posits the existence of a single, central-clock. In that framework, temporal distortions in perception are explained by contracting or expanding time over a given interval. We here present evidence for an alternative account, one which proposes multiple independent timelines coexisting within the brain and stresses the importance of motor predictions and causal inferences in constructing our temporal representation of the world. Participants judged the simultaneity of a beep and flash coming from a single source at different distances. The beep was always presented at a constant delay after a motor action, while the flash occurred at a variable delay. Independent shifts in the implied timing of the auditory stimulus toward the motor action (but not the visual stimulus) provided evidence against a central-clock model. Additionally, the hypothesis that the time between action and delayed effect is compressed (known as intentional binding) seems unable to explain our results: firstly, because actions and effects can perceptually reverse, and secondly because the recalibration of simultaneity remains even after the participant’s intentional actions are no longer present. Contrary to previous reports, we also find that participants are unable to use distance cues to compensate for the relatively slower speed of sound when audio-visual events are presented in depth. When a motor act is used to control the distal event, however, adaptation to the delayed auditory signal occurs and subjective cross-sensory synchrony is maintained. These results support the hypothesis that perceptual timing derives from and is calibrated by our motor interactions with the world.

## Introduction

Brains collect information about the external world through a variety of sensory systems. However, due to differences in transmission velocities, neural architecture, and processing demands, these incoming sensory signals become centrally available to the brain at different points in time (Andreassi and Greco, 1975; Allison et al., 1977; King and Palmer, 1985; Meredith et al., 1987; Spence and Squire, 2003; King, 2005; Eagleman, 2008). The discrepancies in processing times, occurring on the range of tens of milliseconds, have real-world implications. For example, when sprinters line up for the beginning of a race, a gunshot rather than a visual event is used to signal the start of competition. Although sound waves travel much slower than light, auditory information is processed more rapidly in the brain. Sprinters can thus react much faster to a bang than a flash. This behavioral fact has been known for well over a century (Wundt, 1874), and in recent decades has been corroborated by our knowledge of human physiology: the cells in our auditory cortex can change their firing rate more quickly in response to a sound than the visual cortex cells can respond to a light (King and Palmer, 1985).

But comparing the physiology to perception leads to a paradox. While the sprinter can react at different speeds to the incoming sensory information, perceptually the flash and the bang of the pistol seem to occur at the same time. Even more striking, for the official pulling the trigger, the action itself, and even the decision to act, will also appear synchronous with the sight and sound of the gunshot. The volitional and motor signals, generated far in advance of the sensory effects, are brought into perceptual alignment to produce a unified and coherent temporal experience. This fact is all the more perplexing given that humans are capable of detecting differences in timing as small as 2 ms (Wertheimer, 1912; Hirsh and Sherrick, 1961; Westheimer and McKee, 1977), far below the relevant sensory processing delays. What accounts for the sleight of hand that allows perception to rewrite the timing of its outgoing motor acts and incoming sensory feedback?

In interactions with the world, one of the fundamental challenges animals face, crucial both for learning and survival, is that of determining causality (Michotte, 1963; Waldmann and Holyoak, 1992; Buehner and Cheng, 1997; Pearl, 2000; Scholl and Tremoulet, 2000; Eagleman and Holcombe, 2002; Schulz and Gopnik, 2004; Griffiths and Tenenbaum, 2005; Sloman, 2005; Stetson et al., 2006; Körding et al., 2007). At its most fundamental level, causality requires regularity in temporal order judgments; concluding that event B consistently followed action A. Correctly judging the order of action and sensation, however, is not an easy task for the brain to solve, in part because sensory-motor delays are constantly shifting in relation to one another. For example, as limbs grow throughout development, more time is required for motor commands to travel out and for sensory data to return (Campbell et al., 1981; Alison et al., 1983). Changing lighting conditions, such as entering a dimly lit room, cause signals from the retina to be delayed by up to 100 ms (Matteson, 1971; Purpura et al., 1990). Different acoustic environments can modulate the perceived arrival time of sounds (Kinsler et al., 2000). To account for these changing latencies and ensure proper judgments of causality, the brain must be able to dynamically adjust its expectations about the temporal relationship between motor output and incoming sensations (Stetson et al., 2006; Eagleman, 2008).

While it is clear that it would be useful to calibrate the timing of motor acts and sensory feedback, the mechanism by which this is accomplished is not well understood. How would the nervous system know exactly when to calibrate and under what conditions? One proposal is that organisms calibrate time perception through their motor interactions with the world (Stetson et al., 2006; Eagleman, 2008). This notion has related roots in the literature on spatial vision (Welch, 1978; Bedford, 1999; O’Regan and Noe, 2001), but has only recently been explored in relation to time. In spatial vision, for example, when participants wear left-right inverting prism glasses, their vision is highly distorted and they are unable to interact appropriately with the world. Objects on the left now appear on the right. However, if the participant is allowed to interact with the world (reach out and touch objects), he adapts such that the object on the left now appears to be on the left again (Kohler, 1951; Welch, 1978; Redding et al., 1992; Redding and Wallace, 2002). In other words, the brain can send motor actions out into the world and use the feedback to calibrate perceptual interpretations of the world. This calibration of vision allows the brain to maintain accurate judgments in varied and varying environments.

Analogously, in our framework, an animal can send out a motor action (say, snapping one’s fingers) and analyze the returning sensations (the resulting feel, sight, and sound) to calibrate the timing of different modalities. If the animal’s brain were to employ the simple prior expectation that sensations should follow actions without delay, then any sensation arriving at a delay could be brought into temporal alignment. For example if finger-snaps were consistently followed immediately by the feel and sight of the fingers, but the “click” sound came 100 ms later, auditory processing could be adjusted until the click was perceived as synchronous with the other modalities. The temporal alignment of modalities can subsequently be useful during passive viewing of the world.

Crucial to this equation will be an animal’s ability to recognize which changes in the world it is authoring. A “click” sound unrelated to the fingers, but instead indicative of a separate event, does not need to be brought into temporal alignment. According to several influential theories of motor control, it is our ability to monitor self-generated actions that allows us to distinguish the sensory consequences of our own actions from externally produced sensory stimuli (von Holst, 1954; Jordan and Rumelhart, 1992; Jeannerod, 1997; Wolpert and Ghahramani, 2000). This monitoring is carried out by a predictive forward model that can anticipate and identify the sensory consequences of our own movements. A comparison between predicted and actual sensory feedback, carried out by a central monitor (Frith, 1992), is what then allows us to recognize motor actions as our own. Identifying that the delayed “click” has the anticipated sound of fingers being snapped is what licenses the brain to claim authorship and shift the perceived time of the sound closer to the causally related touch, sight, and action.

As the philosopher David Hume pointed out, making these types of causal inferences relies upon three empirical cues: temporal priority, constant conjunction, and contiguity in space and time (Hume, 1748). Experimental results have suggested that the manipulation of any of these cues can profoundly alter the consequent perceptual experience. For example, precise predictions about the tactile feedback, both in time and space, are what prevent us from having the capacity to tickle ourselves (Weiskrantz et al., 1971). However, if the predictability is manipulated, for example by injecting a temporal delay between the motor action and the end effect, participants can be fooled into thinking that another person is tickling them (Blakemore et al., 1999). Ratings of the intensity of the ticklish sensation vary as a function of the ability of the motor command to predict precisely the spatial and temporal position of the resulting sensory feedback (Blakemore et al., 2000).

**Figure 1:**
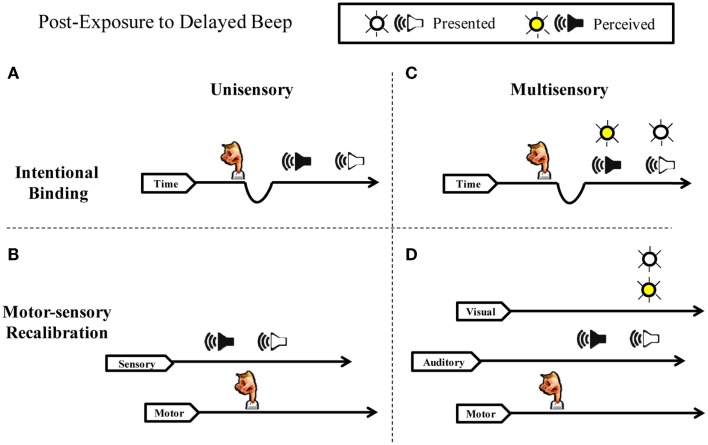
**Intentional binding vs. motor-sensory recalibration**. Schematic illustration of perceived timing following adaptation to a repeated voluntary action and a delayed auditory effect. **(A)** Intentional Binding suggests sensory consequences are pulled toward intentions and that the phenomenon is explained by the slowing of an internal clock. Delayed auditory effects are drawn closer to actions because subjective time contracts. **(B)** Motor-sensory Recalibration proposes multiple independent timelines and highlights the flexibility and uncertainty inherent in a causal understanding of the world. The theory predicts an illusory reversal of action and effect in the unisensory case. **(C)** If subjective duration contracts between actions and effects, simultaneously presented auditory and visual stimuli should shift in unison toward the action. Therefore, simultaneity judgments between the beep and the flash should not change in relation to one another. **(D)** Contrary to the intentional binding model, motor-sensory recalibration predicts an *independent* auditory temporal shift in multi-sensory timing. The timing of the flash does not shift because it is not presented at a predictable delay, but instead varies in time.

Consistent with the hypothesis of recalibration in the time domain, a rich body of experimental work has recently demonstrated that the perceived duration between a repeated voluntary action (pressing a key) and a delayed sensory effect (e.g., seeing a flash or hearing a beep) is contracted (for reviews see Buehner, 2010; Moore and Obhi, 2012). Two competing hypothesis have arisen to explain these results. The first, intentional binding, proposes that the brain “contains a specific cognitive module that binds intentional actions to their effects to construct a coherent conscious experience of our own agency” (Haggard et al., 2002). In this framework, sensory effects are subjectively “pulled” toward intentions, such that all sensations following voluntary action appear closer together in time to the actions (Figure 1A). This “binding” putatively results from a compression of the perceived time between action and sensation, typically explained by variations in the rate of pacing signals from an internal clock mechanism (Wenke and Haggard, 2009). The durations between action and sensation appear shorter, on this account, because fewer clock ticks accumulate during a given interval. Crucially, slowing of an internal clock depends on a close association between a participant’s intentions and the resulting sensory feedback (Haggard et al., 2002; Haggard and Clark, 2003; Wohlschlager et al., 2003; Engbert and Wohlschläger, 2007; Engbert et al., 2008; Moore and Haggard, 2008).

The second account suggests that because of uncertainty (i.e., measurement noise) associated with temporal judgments, estimates of causally related events are more likely to be judged close in time and space than unrelated events (Eagleman and Holcombe, 2002; Stetson et al., 2006; Buehner and Humphreys, 2009, 2010; Buehner, 2010, 2012). This theory accords with the results from intentional binding [e.g., people are most confident that events caused by themselves are causally related (Stetson et al., 2006)], but also predicts attraction for causally linked events outside of one’s own control (Eagleman and Holcombe, 2002). Recent experimental work has supported that hypothesis, showing timing shifts when observing another person perform a causal action (Wohlschlager et al., 2003; Engbert and Wohlschläger, 2007), for joint causal actions (Strother et al., 2010; Obhi and Hall, 2011), for intra-sensory and cross-sensory causally linked events (Haggard and Clark, 2003; Stetson et al., 2006), for non-intentional mechanical causation (Buehner, 2012), and spatial causal binding when no motor planning or intentional action is present (Buehner and Humphreys, 2010; but see Cravo et al., 2009).

Because of the importance of voluntary actions in causal inferences, our hypothesis stresses an active recalibration of the expected timing relationships between outgoing motor acts and resultant sensory signals (Stetson et al., 2006). In this motor-sensory recalibration hypothesis, timing expectations in different modalities (e.g., sensory and motor systems) can shift in relation to one another. In other words, the expectations of how long an action should take to go out, and the expectation of how long sensory feedback should take to return, undergoes dynamic adjustment based on interaction with the world. The injection of a delay violates the expectation that causally related sensory events should occur without delay, and therefore the timing of the system shifts. Stetson et al. (2006) illustrated a striking prediction of this theory: after adaptation to a delay between a button press and flash, a flash presented immediately (and unexpectedly) after a button press will seem to occur *before* the action itself (Figure 1B). It is crucial to note this illusory reversal of action and sensation is incompatible with the intentional binding framework: effects “bound” to their intentions would not occur before the intentions themselves; instead, they would merely draw closer together in time. Similarly, it is difficult to see how a clock-rate model could account for a subjective interval turning negative. Despite these concerns, intentional binding and the clock-rate model remain a common interpretation of the phenomenon (see Moore and Obhi, 2012).

In the present study we perform a series of experiments to distinguish between these two hypotheses. Specifically, we test whether recalibration can occur separately and independently along different sensory channels. Studies in this field have generally focused on perceptual timing when a single uni-modal event follows a motor action (Haggard et al., 2002; Haggard and Clark, 2003; Wohlschlager et al., 2003; Stetson et al., 2006; Engbert et al., 2007, 2008; Moore and Haggard, 2008; Heron et al., 2009; Sugano et al., 2010). While there are numerous studies on cross-modal recalibration (Spence and Squire, 2003; Sugita and Suzuki, 2003; Fujisaki et al., 2004; Vroomen et al., 2004; Navarra et al., 2009, 2007; Hanson et al., 2008; Keetels and Vroomen, 2008), only one to our knowledge has examined cross-sensory timing adaptation when a participant’s own motor actions are involved (Cravo et al., 2011). We reason that if subjective duration contracts between actions and effects (intentional binding), then simultaneously presented auditory and visual stimuli should shift in unison toward the action (Figure 1C), and therefore simultaneity judgments between the beep and the flash should be unchanged in the presence or absence of the motor action. On the other hand, if motor output calibrates timing expectations for vision and audition independently (motor-sensory recalibration model), then these senses will change their perceived timing relationship with each other – but only when a participant’s own motor actions trigger the events (Figure 1D). To distinguish these outcomes, we had participants judge the simultaneity of audio-visual pairings in active and passive conditions – that is when the participant triggers a beep and flash with a key press, or the computer triggers the events.

Additionally, we had participants make simultaneity judgments at different distances from the stimuli. At distances greater than 30 m, sight and sound appear unsynchronized (when you observe a woodchopper at a distance, the fall of the axe appears to precede the sound) – but an unexplored question is this: if you consistently controlled the distant woodchopper with your own motor action, would that cause the sight and sound to become perceptually synchronized? Note this is a simple but novel paradigm that has no embodiment in the natural world: normally, objects beyond your arms reach (and especially at a distance greater than 30 m) are beyond operant control. In this study we leverage operant interactions with distant objects to unmask how sensory signals are integrated normally; this also allows us to address an unresolved debate concerning how distance cues are utilized in perceptual judgments (Engel and Dougherty, 1971; Stone et al., 2001; Spence and Squire, 2003; Sugita and Suzuki, 2003; Kopinska and Harris, 2004; Lewald and Guski, 2004; Alais and Carlile, 2005; Arnold et al., 2005; Harrar and Harris, 2005; Heron et al., 2007).

## Materials and Methods

### Stimuli

The testing apparatus consisted of a wireless trigger device (transmitter) and a wireless stimulus device (receiver) that triggers an independently timed flash and beep. We call this apparatus the “clapboard,” named after the device used in the movie industry to produce simultaneous visual and auditory events for later synchronization. The clapboard’s transmitter, which was connected to the testing computer, was responsible for wirelessly sending the stimulus “go” code along with the stimulus parameters on each trial. This was accomplished by a signal sent from the computer to the transmitter (in the *Passive* condition), or by a push-button attached to the transmitter itself (in the *Active* condition).

The clapboard’s receiver consisted of a microcontroller capable of wireless transmission. The microcontroller was also connected to a LED light and a speaker (model: Event 20/20BAS, 260 mm × 375 mm × 310 mm), which it was responsible for controlling. Both the microcontroller and LED flash sat atop the speaker. These “real-world” stimuli (i.e., an actual flash and bang at a distance) circumvented a confound inherent in some previous studies (Dixon and Spitz, 1980; Sugita and Suzuki, 2003), in which participants wore headphones while watching a visual display at a distance. That can be a problem because it introduces pollution from a related effect, “spatial ventriloquism” (Spence and Squire, 2003; Zampini et al., 2003).

**Figure 2:**
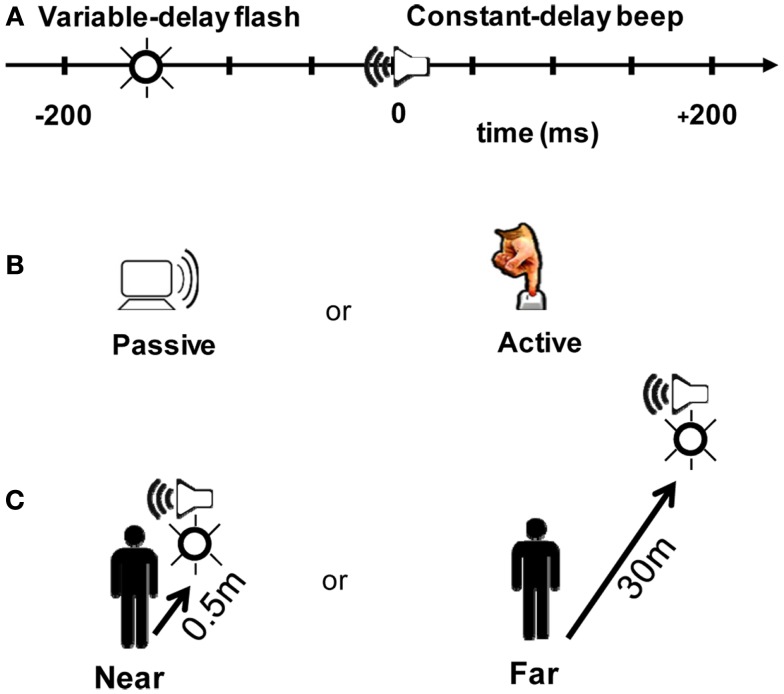
**Experimental design**. **(A)** Participants judged whether a beep and a flash were simultaneous or successive. The beep always arrived at the participant ∼210 ms after a trigger signal was received at the “clapboard,” a device which generates a beep and a flash. On a given trial, a flash could occur within a 200 ms window around the beep at nine possible offsets (multiples of 50 ms). Zero millisecond corresponds to when the beep and the flash physically arrived at the participant at the same time. **(B)** The beep/flash pair was either presented randomly in a 4 s window after the last response (Passive), or triggered by the participant by a button press (Active). **(C)** The beep and the flash were presented at two different distance conditions: Near (clapboard receiver located 0.5 m from participant) or Far (30 m from participant). Stimulus arrival times at the participant were held constant in the Near and Far conditions (i.e., the slower speed of sound was taken into account so that offsets represent the physical arrival time of the signals at the participant).

Each trial consisted of a flash (∼650 cd, 30 ms duration) followed or preceded by a beep (80 dB, 550 Hz, 30 ms duration). Following the trigger signal, the beep arrived at the participant’s ears at a fixed delay of ∼210 ms (200 ms + wireless latency, taking into account the speed of sound as a function of stimulus distance). The wireless turnaround transmission latency (from button press to triggering the stimulus) was <15 ms (<8 ms one-way). The flash occurred from 200 ms before (referred to as “−200” ms) to 200 ms after the beep in 50 ms increments (nine possible stimulus combinations; Figure 2A).

### Procedure

Fifteen trials were recorded at each offset pairing, yielding a total of 135 stimulus presentations per block. The ordering of trials was randomized for each participant.

Participants ran both a Passive and an Active block (Figure 2B). In the Passive block, the beep and the flash occurred randomly within a 4 s window following a participant’s answer from the previous trial. In the Active block, participants triggered an event using a push-button that wirelessly transmitted a signal to the clapboard. Immediately following the cross-modal event, participants judged whether the beep and the flash were simultaneous or successive by recording their response on a keypad. The distribution of the relative timing between the flash and the beep were identical in the two blocks – the only difference is that the flash/beep was a direct result of the participant’s motor act in the operant (Active) case.

We also tested two distance conditions. In the Near condition (0.5 m, Figure 2C), participants were seated in a psychophysical testing room. Light levels were normalized to match the luminance of a lit corridor used in the Far condition (30 m, Figure 2C). The corridor in the Far condition afforded abundant visual and auditory cues for estimating distance. The flash luminance was ∼650 cd; both the perceived luminance and size of the flash were matched between the two distance conditions. Sound volume was also matched to ∼80 dB.

Before running the experiment, participants were required to pass a training version of the Passive task. They then completed a *Passive* block of trials (Block 1), followed by an *Active* block second. We fixed this order of presentation because our initial pilot experiments demonstrated that motor-sensory timing recalibration from an *Active* block can carry over for tens of trials into a subsequent *Passive* block. This aftereffect will be demonstrated and quantified by our experiments below, in which we had a subset of participants complete a third block, this time in the *Passive* condition (Figure 5). That third block will allow us to independently investigate the persistence of aftereffects in the absence of action.

### Participants

At each distance condition [Near (0.5 m) or Far (30 m)], a set of 18 participants participated in both Passive and Active blocks (Figure 2). Six of the participants participated at both distance conditions. Additionally, 10 of the participants from the Near condition completed a third block (Passive) to assess the persistence of adaptation effects (Figure 5). Participants were between the ages of 18 and 45 with normal or corrected-to-normal vision and no known hearing loss. All participants consented to the study as approved by the Institutional Review Board at Baylor College of Medicine, and were compensated for taking part in the experiment.

## Results

### Motor-sensory recalibration

#### Shift in the auditory timeline

**Figure 3:**
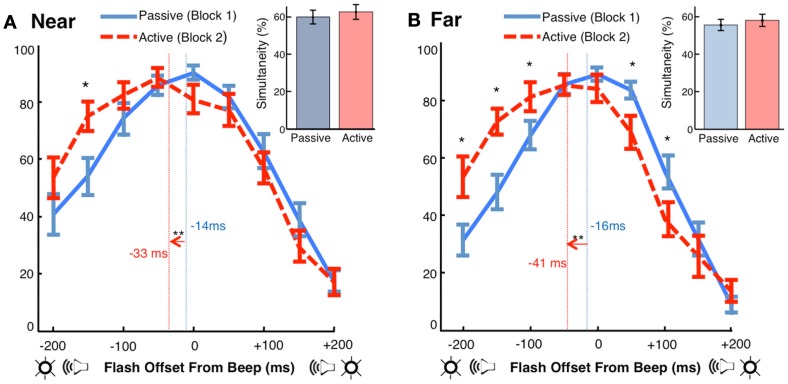
**Motor-sensory recalibration**. **(A)** In the Near condition, the point of subjective simultaneity (PSS, calculated as the center of mass of the simultaneity curves) was −14 ms ± 4 (Passive) and −33 ms ± 5 (Active). The auditory stimulus was perceived as occurring earlier in time in the Active condition by approximately 18 ms [*t*(17) = −3.50, *p* < 0.01]. The difference in the number of simultaneity judgments between conditions was not significant [*t*(17) = 0.84, *p* = 0.41] **(B)** In the Far condition, the PSS was −16 ms ± 4 (Passive) and −41 ms ± 7 (Active). The auditory stimulus was perceived as occurring earlier in time in the Active condition by approximately 25 ms [*t*(17) = −4.04, *p* < 0.01]. The difference in the number of simultaneity judgments between conditions was not significant [*t*(17) = 1.11, *p* = 0.28].

Using simultaneity judgments as a measure of cross-modal timing, we found a replication of the phenomenon of motor-sensory recalibration: a beep occurring at a predicable delay of 210 ms after a motor action was perceived as occurring earlier in time (Figures 3A,B). Shifts in participants’ points of subjective simultaneity (PSS) between the Active and Passive conditions were −18 ms [*t*(17) = −3.50, *p* < 0.01] in the Near location and −25 ms [*t*(17) = −4.04, *p* < 0.01] in the Far location. These are comparable to what has been observed in previous experiments with single modality events following motor actions (Haggard et al., 2002; Stetson et al., 2006) and parallel the results obtained in a recent study of motor-triggered cross-sensory timing (Cravo et al., 2011). However, we note that methodological differences between the Cravo et al. (2011) paper and our study, including explicit adaptation to the action event (instead of our implicit method), potential aftereffects induced by mixed ordering of conditions (instead of our fixed ordering), and a longer interval between action and sensory consequences (300 vs. 210 ms, *personal communication*), may have contributed to differences in the size and nature of the effect across the two studies.

Notably, the shifts in our experiment were not accompanied by a significant difference between the number of simultaneity judgments made in the Active and Passive conditions (Figures 3A,B *inset*). Simultaneity judgments, while limiting the effects of response bias, are susceptible to changes in criterion for what is classified as simultaneous (Zampini et al., 2005; van Eijk et al., 2008; Spence, 2010). Because the effect in our experiment is primarily a lateral shift in the curve (i.e., total simultaneity judgments did not change), a criterion bias cannot explain these results.

#### Increased simultaneity immediately following the motor act

Although we have made the argument that a shift of the auditory timeline best explains our findings (in other words, recalibrated expectations of the timing of the beep), we also noted that in the 150 ms immediately following action, changes in simultaneity judgments between the Passive and Active conditions were much larger than those in the corresponding last three offsets (note the larger separation of the *Passive* and *Active* curves on the left side vs. the right side; Figures 3A,B). This led us to reason that for equally large offsets between flash and beep (e.g., −150 or +150 ms), proximity to the motor act may have influenced the perception of simultaneity of the two sensory events. We now turn to two possible explanations for this asymmetry.

Many studies of motor-sensory recalibration have shown that the strength of the shift between action and effect dissipates with longer delays between action and feedback (Eagleman and Holcombe, 2002; Haggard et al., 2002; Stetson et al., 2006; Heron et al., 2009; Cravo et al., 2011; Arnold et al., 2012; but see Humphreys and Buehner, 2009). Relatedly, Wenke and Haggard (2009) have provided evidence that participants are more likely to judge two tactile events as simultaneous when they are presented within a 150 ms window after a key press; there is no effect for events occurring later than this window. According to Wenke and Haggard (2009), recalibration models cannot account for this data; instead, it is viewed as evidence in favor of a clock-rate model. In their view, intentional actions “transiently slow down an internal clock” and “two shocks are thus more likely to fall within a single clock period, impairing temporal discrimination” (Wenke and Haggard, 2009). We suggest an alternative interpretation of these results that both accords with the motor-sensory recalibration framework and is supported by our present data in Figure 3.

In addition to having a prior expectation that the sensory consequences of actions should occur without delay (Stetson et al., 2006), we hypothesize that the perceptual system also interprets events occurring at short delays after an action as sensory consequences of the action (Hume, 1748; Eagleman and Holcombe, 2002). Moreover, if participants believe that two sensory events originate from a common source, they are more likely to perceive those events as simultaneous with one another (Zampini et al., 2005; van Wassenhove et al., 2007; Stevenson et al., 2012) – in the context of the current study, the common source is their own action. Therefore, we hypothesize that two sensory events closely following a motor act are more likely to be interpreted as (1) caused by the agent, and (2) simultaneous with one another.

Studies of intentional binding have suggested that changes in the timing of sensory events are driven by both a predictive motor component (Haggard et al., 2002; Stetson et al., 2006) and a postdictive inferential mechanism (Moore and Haggard, 2008; Buehner, 2010). These two information sources both contribute to conscious awareness and appear to be weighted in a Bayesian manner according to their reliability. In our experiment, relatively higher rates of simultaneity were observed when the beep and the flash occurred in close proximity to the motor act, paralleling the findings of Wenke and Haggard (2009). Due to its unpredictability, the flash was presumably not subject to motor-sensory shifts (Cravo et al., 2011). Rather, we propose that when the flash occurred shortly after the motor act, postdictive inferential mechanisms linked both sensory events to the action, thereby leading to increased simultaneity judgments. The longer the delay between a button press and sensory feedback, the less likely the brain is to claim authorship over the event and judge the two events as simultaneous (Eagleman and Holcombe, 2002).

### Simultaneity constancy

#### No compensation for distance from the participant

**Figure 4:**
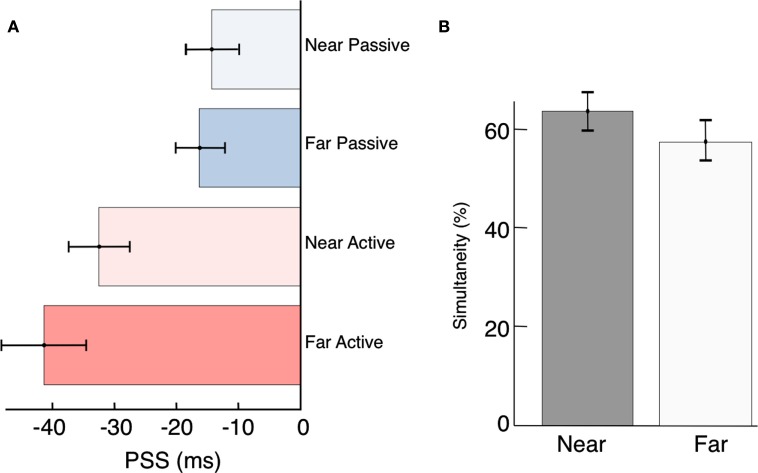
**Compensation for source distance?** **(A)** Participant’s PSS were not significantly different between the near and far conditions [Passive, *t*(34) = −0.36, *p* = 0.72; and Active, *t*(34) = −1.04, *p* = 0.30]. However, active control over a distal event can compensate for distance-induced auditory delays [*t*(17) = −4.04, *p* < 0.01]. **(B)** Although it was not significant, we did observe a trend toward increased overall simultaneity at the Near location. We speculate that this may be driven by the ease of source localization when the stimuli are presented up close, and could be clarified with a higher sample size in the future.

Contrary to previous reports (Sugita and Suzuki, 2003; Kopinska and Harris, 2004; Alais and Carlile, 2005) we find no evidence for compensation of auditory travel time when stimuli are presented at different distances. Expressed as arrival time at the participant’s sensory organs, the PSS was −14 ms in the Near Passive condition and −33 ms in the Near Active condition (Figure 4A). If participants were able to judge the timing of the events as they are leaving the source, the PSS should have shifted to the left in both of the Far conditions by approximately 87 ms (sound takes ∼87 ms to travel 30 m). Thus, compensation for distance-induced auditory delays would have predicted a PSS of −101 ms (−14–87 ms) in the Far Passive condition, and −120 ms in the (−33–87 ms) in the Far Active condition. Instead, we found that stimulus travel times map nearly perfectly onto perceptual time. We will return to this point below, in the Discussion.

#### Does uncertainty in source localization decrease judgments of simultaneity?

Previous studies have suggested that localization and synchrony judgments are dependent on the spatial and temporal properties of the stimulus (Bertelson and Aschersleben, 2003; Hairston et al., 2003; Alais and Burr, 2004; Bertelson and de Gelder, 2004; Zampini et al., 2005; Körding et al., 2007; Shams and Beierholm, 2010; Heron et al., 2012; Stevenson et al., 2012). In our results a relatively higher (although non-significant) number of overall simultaneity judgments were made when participants were seated close to the stimuli (Figure 4B). Although no explicit measures of localization were recorded, we suspect this difference occurred because participants had abundant auditory and visual cues with which to localize the sensory events in the Near condition. They could thus be certain that both the beep and the flash were emanating from the same source (Spence et al., 2003; Gepshtein et al., 2005; Körding et al., 2007; van Wassenhove et al., 2007; Shams and Beierholm, 2010). The greater distance of 30 m may have increased the uncertainty associated with participants’ localization judgments. While studies have shown contraction of spatial locations for causally related events (Buehner and Humphreys, 2010), the influence of distance on judgments of causality (and hence simultaneity) is a largely unexplored question and will be investigated in future studies.

### Perceptual aftereffects

If brains calibrate time perception primarily through motor interaction with the world (see Introduction) one might expect the effects of adaptation to a fixed delay to persist when that delay is taken away (Cunningham et al., 2001; Kennedy et al., 2009). Indeed, the illusory reversal of action and effect (Stetson et al., 2006) is made possible by just such persistence. However, the way in which temporal judgments are affected when the motor act itself is removed following adaptation in unknown. Studies of adaptation to spatial misalignment (Redding and Wallace, 1993), as well as recalibration with inter-sensory stimuli (Fujisaki et al., 2004), suggest that residual perceptual aftereffects might exist. To address this possibility, we had a subset of our participants (*n* = 10) run an additional Passive block (Near condition) following the Active block.

**Figure 5:**
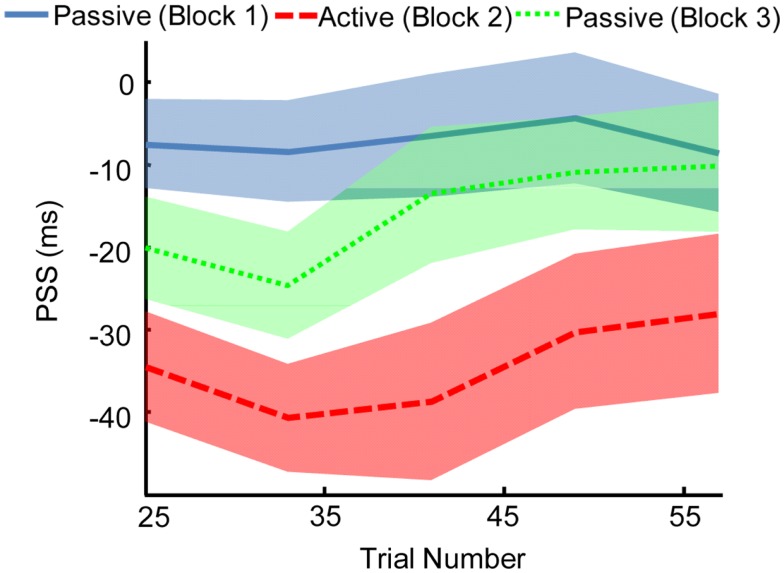
**The effect of motor-sensory recalibration persists in a subsequent passive Block**. This aftereffect lasts for ∼35–40 trials. Point of subjective simultaneity (PSS) was calculated over a window size of 25 trials, with the window shifting in increments of 8 trials. Width of line indicates SEM.

We found that the effects of motor-sensory recalibration remain for ∼35 trials before dissipating (Figure 5). The uncoupling from the motor timeline seems to allow the auditory and visual timelines to shift fairly quickly back into alignment. This finding has parallels in the rapid pace at which motor-sensory recalibration establishes itself, reaching full magnitude within ∼20 trials (Stetson et al., 2006). The speeds with which these shifts in timing take place illustrate the central role of causality in our perceptual interpretation of the world. In fact, recent experiments on cross-sensory recalibration have found shifts following exposure to a single presentation of only a few milliseconds (Wozny and Shams, 2011).

## Discussion

The results of our experiments yield three insights. First, the shift in the timing of the auditory stimulus in relation to the visual stimulus contradicts previous explanations of intentional binding, particularly a clock-rate model, and instead supports the hypothesis of multiple coexisting timelines in the brain. Second, motor-sensory recalibration seems to be driven by both predictive motor signals and postdictive inferential mechanisms. Because of constantly changing neural delays and the critical importance of uncovering causal relationships, the brain utilizes a flexible and adaptive mechanism, rather than simple neural latencies, to construct the timing of events. Third, changes in observer-stimulus distance, resulting in differences in the arrival time of sight and sound, are not taken into account when participants make simultaneity judgments. However, active control over a distal event can result in compensation for the slower speed of sound.

In addition, we tentatively suggest that differences in source localization between the Near and Far conditions may have affected the size of the window that people use to make judgments of synchrony.

### Time to throw out the clock?

For over 50 years the dominant paradigm in time perception research has posited the existence of a single, central-clock responsible for constructing a single temporal representation of the outside world (Creelman, 1962; Treisman, 1963; Allan and Kristofferson, 1974; Gibbon et al., 1984). Distortions in duration and timing are accounted for by increasing or decreasing the amount of “ticks” that accumulate during a given interval. Despite an absence of physiological evidence, a majority of findings in the field still rely on this putative clock to explain their results (Wittmann, 1999; Hodinott-Hill et al., 2002; Tse et al., 2004; Morrone et al., 2005; Kanai et al., 2006; Wearden, 2008; New and Scholl, 2009; Wencil et al., 2010). In the intentional binding literature, performing an action is said to “slow down an internal clock, in anticipation of the effect of the action” (Wenke and Haggard, 2009). In other words, because of fewer clock cycles, the interval between action and effects becomes compressed.

Such a model is incapable of explaining the results of our experiment. If a compression of the interval separating action and effect were responsible for the shift in motor-sensory timing, the beep and the flash would both have shifted toward the motor act by the same amount (Figure 1C), perhaps resulting in more synchrony in the Active condition. That is, a single slowed clock would affect both sensory consequences equally. Instead, we found that different sensory modalities were able to shift individually in relation to the motor act, and the propensity of participants to make judgments of simultaneity was unaltered between the Passive and Active conditions. Replicating previous studies (Haggard et al., 2002), an auditory stimulus occurring at a predictable delay was perceived as occurring closer in time to the action which caused it. This shift occurred, however, without a concomitant change in the timing of the paired visual stimulus. Rather than time itself being stretched or shrunk, the sensory signals themselves were realigned in subjective time. We also suggest that such a process operates implicitly and below the level of awareness. Participants questioned after the Active block reported being unaware of which stimuli was occurring at a constant delay.

We propose that our results are best explained by an appeal to multiple representations of time that coexist within the brain. Trapped by the assumption of a Cartesian theater in which sensory input is passively recorded (Dennett and Kinsbourne, 1992), modern theories of brain time have largely avoided this framework. Mounting evidence, however, suggests that a single clock-rate model of perceptual time is untenable (Eagleman, 2008). Instead, different aspects of time appear to be underpinned by separate neural mechanisms that sometimes act in concert, but are not required to do so (Eagleman and Pariyadath, 2009).

Previous work has provided compelling evidence for the existence of independent motor and sensory timelines in the brain (Ivry, 1996; Ivry and Richardson, 2002; Stetson et al., 2006; Arnold and Yarrow, 2011). The current experiment extends these findings and shows that individual sensory modalities have their own adjustable timelines. If each sense calibrates against motor acts, this calibrates them in relation to each other as well. Neither the illusory reversal of action and effect (Stetson et al., 2006), nor the sensory specific modulation of cross-modal simultaneity observed here can be explained by a clock-rate model. In light of evidence from other labs (Westheimer, 1999; Yarrow et al., 2004; Morrone et al., 2005; Johnston et al., 2006; Burr et al., 2007; van Wassenhove et al., 2008; Alais and Cass, 2010; Marinovic and Arnold, 2012), we suggest that a paradigm shift is underway within the field of time perception. Discarding the notion of a single central timer allows for novel frameworks and predictions (Westheimer, 1999; Körding et al., 2007; Ivry and Schlerf, 2008; Buhusi and Meck, 2009; Buonomano and Maass, 2009; Eagleman and Pariyadath, 2009; van Wassenhove, 2009; Johnston, 2010; Ahrens and Sahani, 2011; Cai et al., 2012; Liverence and Scholl, 2012; Pacer and Griffiths, 2012) that will force us to think critically about what it means for time to be represented in the brain.

### Actions calibrate time perception

Experiments have suggested that our experience of the temporal properties of an event are a result of both predictive (Stetson et al., 2006) and postdictive or inferential mechanisms (Eagleman and Sejnowski, 2000). Retrospective awareness has been reported for both sensory (Choi and Scholl, 2006) and motor (Moore and Haggard, 2008) events and seems to operate over a window 250 ms into the future (Moore et al., 2009). In our experiment, the largest shifts between Passive and Active blocks occurred in the 150 ms immediately following the action. A similar effect, increased simultaneity judgments of two tactile stimuli in a window 150 ms after an action, has been taken as decisive evidence against the motor-sensory recalibration model (Wenke and Haggard, 2009). The authors assume that recalibration only affects when in time events occur, as opposed to affecting the judgment criteria for synchrony. That explanation ignores the causal component that serves as the foundation of our framework. Instead of a slowed clock, we suggest that when a beep and the flash occur in a brief window after the motor action, the brain becomes more likely to claim authorship over the sensory consequences. Because of a prior assumption that sensory consequences of an action should arrive without delay, events causally related to the action are more likely to be judged as simultaneous. Crucially, the influence of the motor signal is limited by its predictive ability, which decays over time.

We have previously suggested that the brain must continually refine its expectations about the normal temporal relationship between outgoing actions and incoming sensations (Stetson et al., 2006; Eagleman, 2008). In this framework, motor interaction with the world calibrates expectations about the timing of feedback from the different sensory channels. These expectations about sensory timing can subsequently be employed when passively interpreting events in the world (i.e., events that were not self-caused). This theoretical framework explicitly predicts that perceptual aftereffects should be observed even in the absence of action, and this is indeed what we found (Figure 5). While these temporal aftereffects are not necessarily inconsistent with an intentional binding model, they are an unambiguous prediction of the motor-sensory recalibration model (Stetson et al., 2006; Eagleman, 2008; Cai et al., 2012). Note that the aftereffects we found only lasted ∼35–40 trials into the Passive block; we hypothesize this could be extended by longer training in the Active condition, and our future experiments will test this prediction. Finally, it is interesting to note that our results appear similar, at least on the surface, to reaching aftereffects observed following exposure to spatial misalignment during prism adaptation (Redding et al., 2005). The links between these two research traditions (recalibration to misalignment in time or in space) has only begun to be investigated (Kennedy et al., 2009; Cai et al., 2012) and more studies are needed to elucidate common principles and interactions.

### Actions compensate for distance-induced auditory delays

Our results present a picture in which active control over a distant audio-visual event can modulate its perceived simultaneity. A person observing fireworks at a distance of 30 m (Far condition) would notice a temporal asynchrony between the bang and flash, due to the slower velocity of sound. If that same observer were given a chance to control the onset of the fireworks however, the bang and flash would be more likely to be perceived as a unitary event. Although this appears to support the hypothesis that brains can compensate for delays in auditory travel times, the mechanism is different from that originally proposed.

Beginning with Engel and Dougherty (1971), several studies have suggested that the brain is able to integrate information about distance (whether visual, auditory, or both) to calibrate simultaneity (Sugita and Suzuki, 2003; Kopinska and Harris, 2004; Alais and Carlile, 2005). The temporal location of an integration window is purportedly actively manipulated by the brain depending on the distance of the visible sound source (Spence and Squire, 2003; Sugita and Suzuki, 2003). The window does not widen in size but rather shifts along a timeline. Some authors have interpreted this as reflecting a perceptual mechanism similar to size constancy, a phenomenon wherein the perceived size of an object is maintained despite variations in the retinal information (Gregory, 1963; Kopinska and Harris, 2004; Harris et al., 2010). Such constancies are common for other perceptual attributes including color, brightness, shape, and location (Palmer, 1999).

Our results conflict with these studies and show that the differential velocities of sound and light map nearly directly onto the perceived timing of audio-visual events (Figure 4). Several other experiments have questioned the notion of active compensation for source distance and our results concord with these studies (Stone et al., 2001; Lewald and Guski, 2004; Arnold et al., 2005; Heron et al., 2007). As others have pointed out, such a mechanism would require calculations utilizing absolute distance as well as the speed of sound in different environmental settings (Arnold et al., 2005; Heron et al., 2007). In addition to the computational complexity of such a task, it is not apparent why the brain would want to explicitly represent such variables in the first place. Methodological differences between the studies, including the use of a binary forced choice task (Sugita and Suzuki, 2003; Kopinska and Harris, 2004; Arnold et al., 2005), sound presentations through headphones (Sugita and Suzuki, 2003), requirements to use one’s imagination (Sugita and Suzuki, 2003), and a lack of physical distance cues (Alais and Carlile, 2005) may have contributed to biased reporting. In line with Arnold et al. (2005), we interpret studies showing active compensation as likely deriving from cognitive strategies tapping into participant’s knowledge about the slower speed of sound in the physical world. The use of simultaneity judgments in our experiment limited the effects of response biases and made any attempt to use a cognitive strategy problematic.

While our results did not provide evidence of compensation for distance-induced auditory delays, we did find differences between the Near and Far conditions in participant’s proclivity to make simultaneity judgments (Figure 4B). Rather than a moveable window shifting along a timeline (Sugita and Suzuki, 2003) our results point toward an integration period that can expand or shrink depending on various spatial and temporal factors of the stimulus. Previous research has suggested that the impression of a plausible unitary event (Guski and Troje, 2003), driven by the temporal synchrony and spatial coincidence of cross-modal stimuli (Körding et al., 2007; van Wassenhove et al., 2007; Shams and Beierholm, 2010; Stevenson et al., 2012), can lead to higher causality ratings and thus increased simultaneity judgments. Our results suggest that differences in source localization caused by changes in distance may also contribute to the perception of a single causal event. Participants were more likely, in both the Passive and Active cases, to judge audio-visual pairings as simultaneous if they were presented directly in front of them (Near condition). Although the brightness of the flash and the loudness of the beep were matched in the Far condition, participants appeared to be less certain that the audio-visual event was emanating from a single location. No other study, to our knowledge, has reported how differences in distance affect the size of the window for cross-modal integration. Future experiments might investigate the flexibility of our causal perception by independently varying the stimulus distances of simultaneously presented auditory and visual stimuli. Such experiments would contribute to a better understanding of the relative roles of perceptual and cognitive factors in our causal judgments.

## Conclusion

The conventional framework for understanding temporal perception has focused on how the brain passively registers a feed-forward flow of sensory input. We suggest instead that the timing of events is actively constructed by the brain through disparate mechanisms which can be teased apart with experimentation. Crucial to this construction is the brain’s ability to distinguish what changes in the environment it is responsible for causing. Because of the difficulty and importance of making such inferences, timing judgments are flexible and dynamically calibrated in order to keep causality assessments accurate. Our motor actions have a special role to play in modulating the expectations associated with sensory feedback and hence perception. While the influence of motor signals on our spatial representation of the world is well established, contributions to temporal perception are still largely unexplored and warrant further investigation.

## Conflict of Interest Statement

The authors declare that the research was conducted in the absence of any commercial or financial relationships that could be construed as a potential conflict of interest.

## References

[B1] Ahrens M. B. Sahani M. (2011). Observers exploit stochastic models of sensory change to help judge the passage of time. Curr. Biol. 21, 200–206 10.1016/j.cub.2010.12.043 21256018 PMC3094759

[B2] Alais D. Burr D. (2004). The ventriloquist effect results from near-optimal bimodal integration. Curr. Biol. 14, 257–262 10.1016/S0960-9822(04)00043-0 14761661

[B3] Alais D. Carlile S. (2005). Synchronizing to real events: subjective audiovisual alignment scales with perceived auditory depth and speed of sound. Proc. Natl. Acad. Sci. U.S.A 102, 2244–2247 10.1073/pnas.0407034102 15668388 PMC548526

[B4] Alais D. Cass J. (2010). Multisensory perceptual learning of temporal order: audiovisual learning transfers to vision but not audition. PLoS ONE 5:e11283 10.1371/journal.pone.0011283 20585664 PMC2890588

[B5] Alison T. Wood C. Goff W. (1983). Brain stem auditory, pattern-reversal visual and short latency somatosensory evoked potentials: latencies in relation to age, sex and brain and body type. Electroencephalogr. Clin. Neurophysiol. 55, 619–636 10.1016/0013-4694(83)90272-9 6189692

[B6] Allan L. G. Kristofferson A. B. (1974). Psychophysical theories of duration discrimination. Atten. Percept. Psychophys. 16, 26–34 10.3758/BF03203244

[B7] Allison T. Matsumiya Y. Goff G. D. Goff W. R. (1977). The scalp topography of human visual evoked potentials. Electroencephalogr. Clin. Neurophysiol. 42, 185–197 10.1016/0013-4694(77)90025-6 65254

[B8] Andreassi J. L. Greco J. R. (1975). Effects of bisensory stimulation on reaction time and the evoked cortical potential. Physiol. Psychol. 3, 189–194

[B9] Arnold D. H. Johnston A. Nishida S. (2005). Timing sight and sound. Vision Res. 45, 1275–1284 10.1016/j.visres.2004.11.014 15733960

[B10] Arnold D. H. Nancarrow K. Yarrow K. (2012). The critical events for motor-sensory temporal recalibration. Front. Hum. Neurosci. 6:235 10.3389/fnhum.2012.00235 22891056 PMC3413957

[B11] Arnold D. H. Yarrow K. (2011). Temporal recalibration of vision. Proc. Biol. Sci. 278, 535–558 10.1098/rspb.2010.1396 20826481 PMC3025680

[B12] Bedford F. L. (1999). Keeping perception accurate. Trends Cogn. Sci. (Regul. Ed.) 3, 4–11 10.1016/S1364-6613(98)01266-2 10234221

[B13] Bertelson P. Aschersleben G. (2003). Temporal ventriloquism: Cross-modal interaction on the time dimension: 1 Evidence from auditory-visual temporal order judgment. Int. J. Psychophysiol. 50, 147–155 10.1016/S0167-8760(03)00130-2 14511842

[B14] Bertelson P. de Gelder B. (2004). “The psychology of multimodal perception,” in Crossmodal Space and Crossmodal Attention, eds Spence C. Driver J. (Oxford: Oxford University Press), 151–177

[B15] Blakemore S. J. Frith C. D. Wolpert D. M. (1999). Spatio-temporal prediction modulates the perception of self-produced stimuli. J. Cogn. Neurosci. 11, 551–559 10.1162/089892999563607 10511643

[B16] Blakemore S.-J. Wolpert D. M. Frith C. D. (2000). Why can’t you tickle yourself? Neuroreport 11, 16 10.1097/00001756-200012180-00006 10943682

[B17] Buehner J. (2010). “Temporal Binding,” in Attention and Time, eds Nobre K. Coull J. (Oxford: Oxford University Press), 201–211

[B18] Buehner M. J. (2012). Understanding the past, predicting the future: causation, not intentional action, is the root of temporal binding. Psychol. Sci. 23, 1490–1497 10.1177/0956797612444612 23104679

[B19] Buehner M. J. Cheng P. W. (1997). “Causal induction: the power pc theory versus the Rescorla–Wagner model,” in Proceedings of the Nineteenth Annual Conference of the Cognitive Science Society (Hillsdale, NJ: Erlbaum), 55–60

[B20] Buehner M. J. Humphreys G. R. (2009). Causal binding of actions to their effects. Psychol. Sci. 20, 1221–1228 10.1111/j.1467-9280.2009.02435.x 19732384

[B21] Buehner M. J. Humphreys G. R. (2010). Causal contraction: spatial binding in the perception of collision events. Psychol. Sci. 21, 44–48 10.1177/0956797609354735 20424021

[B22] Buhusi C. V. Meck W. H. (2009). Relativity theory and time perception: single or multiple clocks? PLoS ONE 4:e6268 10.1371/journal.pone.0006268 19623247 PMC2707607

[B23] Buonomano D. V. Maass W. (2009). State-dependent computations: spatiotemporal processing in cortical networks. Nat. Rev. Neurosci. 10, 113–125 10.1038/nrn2558 19145235

[B24] Burr D. Tozzi A. Morrone M. C. (2007). Neural mechanisms for timing visual events are spatially selective in real-world coordinates. Nat. Neurosci. 10, 423 17369824 10.1038/nn1874

[B25] Cai M. Stetson C. A. Eagleman D. M. (2012). A neural model for temporal order judgments and their active recalibration: proposing a common mechanism for space and time. Front. Psychol. 3:470 10.3389/fpsyg.2012.00470 23130010 PMC3487422

[B26] Campbell W. W. Ward L. C. Swift T. R. (1981). Nerve conduction velocity varies inversely with height. Muscle Nerve 4, 520–523 10.1002/mus.880040609 7311991

[B27] Choi H. Scholl B. J. (2006). Perceiving causality after the fact: postdiction in the temporal dynamics of causal perception. Perception 35, 385–399 10.1068/p5462 16619953

[B28] Cravo A. M. Claessens P. M. Baldo M. V. (2009). Voluntary action and causality in temporal binding. Exp. Brain Res. 199, 95–99 10.1007/s00221-009-1969-0 19680639

[B29] Cravo A. M. Claessens P. M. Baldo M. V. (2011). The relation between action, predictability and temporal contiguity in temporal binding. Acta Psychol. (Amst.) 136, 157–166 10.1016/j.actpsy.2010.11.005 21185547

[B30] Creelman C. D. (1962). Human discrimination of auditory duration. J. Acoust. Soc. Am. 34, 528–593 10.1121/1.1918172

[B31] Cunningham D. W. Billock V. A. Tsou B. H. (2001). Sensorimotor adaptation to violations of temporal contiguity. Psychol. Sci. 12, 532–535 10.1111/1467-9280.00328 11760144

[B32] Dennett D. C. Kinsbourne M. (1992). Time and the observer: the where and when of consciousness in the brain. Behav. Brain Sci. 15, 183–201 10.1017/S0140525X00067339

[B33] Dixon N. F. Spitz L. (1980). The detection of auditory visual desynchrony. Perception 9, 719–721 10.1068/p090719 7220244

[B34] Eagleman D. M. (2008). Human time perception and its illusions. Curr. Opin. Neurobiol. 18, 131–136 10.1016/j.conb.2008.06.002 18639634 PMC2866156

[B35] Eagleman D. M. Holcombe A. O. (2002). Causality and the perception of time. Trends Cogn. Sci. (Regul. Ed.) 6, 323–325 10.1016/S1364-6613(02)01945-9 12140076

[B36] Eagleman D. M. Pariyadath V. (2009). Is subjective duration a signature of coding efficiency? Philos. Trans. R. Soc. Lond. B Biol. Sci 364, 1841–1851 10.1098/rstb.2009.0026 19487187 PMC2685825

[B37] Eagleman D. M. Sejnowski T. J. (2000). Motion integration and postdiction in visual awareness. Science 287, 2036–2038 10.1126/science.287.5460.2036 10720334

[B38] Engbert K. Wohlschläger A. (2007). Intentions and expectations in temporal binding. Conscious. Cogn. 16, 255–264 10.1016/j.concog.2006.09.010 17113309

[B39] Engbert K. Wohlschläger A. Haggard P. (2008). Who is causing what? The sense of agency is relational and efferent-triggered. Cognition 107, 693–704 10.1016/j.cognition.2007.07.021 17825813

[B40] Engbert K. Wohlschläger A. Thomas R. Haggard P. (2007). Agency, subjective time, and other minds. J. Exp. Psychol. Hum. Percept. Perform. 33, 1261–1268 10.1037/0096-1523.33.6.1261 18085941

[B41] Engel G. R. Dougherty W. G. (1971). Visual–auditory distance constancy. Nature 234, 308 10.1038/234308a0 4945010

[B42] Frith C. (1992). The Cognitive Neuropsychology of Schizophrenia. Hove: Lawrence Erlbaum Associates

[B43] Fujisaki W. Shimojo S. Kashino M. Nishida S. (2004). Recalibration of audiovisual simultaneity. Nat. Neurosci. 7, 773–778 10.1038/nn1268 15195098

[B44] Gepshtein S. Burge J. Ernst M. O. Banks M. S. (2005). The combination of vision and touch depends on spatial proximity. J. Vis. 5, 1013–1023 10.1167/5.11.7 16441199 PMC2632311

[B45] Gibbon J. Church R. M. Meck W. H. (1984). Scalar timing in memory. Ann. N. Y. Acad. Sci. 423, 52–77 10.1111/j.1749-6632.1984.tb23417.x 6588812

[B46] Gregory R. L. (1963). Distortion of visual space as inappropriate constancy scaling. Nature 199, 678–680 10.1038/199678a0 14074555

[B47] Griffiths T. L. Tenenbaum J. B. (2005). Structure and strength in causal induction. Cogn. Psychol. 51, 334–384 10.1016/j.cogpsych.2005.05.004 16168981

[B48] Guski R. Troje N. F. (2003). Audiovisual phenomenal causality. Percept. Psychophys. 65, 789–800 10.3758/BF03194815 12956586

[B49] Haggard P. Clark S. (2003). Intentional action: conscious experience and neural prediction. Conscious. Cogn. 12, 695–707 10.1016/S1053-8100(03)00052-7 14656511

[B50] Haggard P. Clark S. Kalogeras J. (2002). Voluntary action and conscious awareness. Nat. Neurosci. 5, 382–385 10.1038/nn827 11896397

[B51] Hairston W. D. Wallace M. T. Vaughan J. W. Stein B. E. Norris J. L. Schirillo J. A. (2003). Visual localization ability influences cross-modal bias. J. Cogn. Neurosci. 15, 20–29 10.1162/089892903321107792 12590840

[B52] Hanson J. V. Heron J. Whitaker D. (2008). Recalibration of perceived time across sensory modalities. Exp. Brain Res. 185, 347–352 10.1007/s00221-008-1282-3 18236035

[B53] Harrar V. Harris L. R. (2005). Simultaneity constancy: detecting events with touch and vision. Exp. Brain Res. 166, 465–473 10.1007/s00221-005-2386-7 16028031

[B54] Harris L. R. Harrar V. Jaekl P. Kopinska A. (2010). “Mechanisms of simultaneity constancy,” in Space and Time in Perception and Action, eds Nijhawan R. Khurana B. (Cambridge: Cambridge University Press), 232–253

[B55] Heron J. Hanson J. V. Whitaker D. (2009). Effect before cause: supramodal recalibration of sensorimotor timing. PLoS ONE 4:e7681 10.1371/journal.pone.0007681 19890383 PMC2766625

[B56] Heron J. Roach N. W. Hanson J. V. McGraw P. V. Whitaker D. (2012). Audiovisual time perception is spatially specific. Exp. Brain Res. 218, 477–485 10.1007/s00221-012-3038-3 22367399 PMC3324684

[B57] Heron J. Whitaker D. McGraw P. V. Horoshenkov K. V. (2007). Adaptation minimizes distance-related audiovisual delays. J. Vis. 7, 1–8 10.1167/7.6.1 17997633

[B58] Hirsh I. J. Sherrick C. E. (1961). Perceived order in different sense modalities. J. Exp. Psychol. 62, 423–432 10.1037/h0045283 13907740

[B59] Hodinott-Hill I. Thilo K. V. Cowey A. Walsh V. (2002). Auditory chronostasis: hanging on the telephone. Curr. Biol. 12, 1779–1781 10.1016/S0960-9822(02)01219-8 12401174

[B60] Hume D. (1748). Enquiry Concerning Human Understanding. New York, NY: PF Collier and Son

[B61] Humphreys G. R. Buehner M. J. (2009). Magnitude estimation reveals temporal binding at super-second intervals. J. Exp. Psychol. Hum. Percept. Perform. 35, 1542–1549 10.1037/a0014492 19803655

[B62] Ivry R. B. (1996). The representation of temporal information in perception and motor control. Curr. Opin. Neurobiol. 6, 851–857 10.1016/S0959-4388(96)80037-7 9000026

[B63] Ivry R. B. Richardson T. C. (2002). Temporal control and coordination: the multiple timer model. Brain Cogn. 48, 117–132 10.1006/brcg.2001.1308 11812037

[B64] Ivry R. B. Schlerf J. E. (2008). Dedicated and intrinsic models of time perception. Trends Cogn. Sci. (Regul. Ed.) 12, 273–280 10.1016/j.tics.2008.04.002 18539519 PMC4335014

[B65] Jeannerod M. (1997). The Cognitive Neuroscience of Action. Oxford: Blackwell

[B66] Johnston A. (2010). “Modulation of time perception by visual adaptation,” in Attention and Time, eds Nobre K. Coull J. (Oxford: Oxford University Press), 187

[B67] Johnston A. Arnold D. H. Nishida S. (2006). Spatially localized distortions of event time. Curr. Biol. 16, 472–479 10.1016/j.cub.2006.01.032 16527741

[B68] Jordan M. I. Rumelhart D. E. (1992). Forward models: supervised learning with a distal teacher. Cogn. Sci. 16, 307–354 10.1207/s15516709cog1603_1

[B69] Kanai R. Paffen C. L. Hogendoorn H. Verstraten F. A. (2006). Time dilation in dynamic visual display. J. Vis. 6, 1421–1430 10.1167/6.12.4 17209745

[B70] Keetels M. Vroomen J. (2008). Temporal recalibration to tactile-visual asynchronous stimuli. Neurosci. Lett. 430, 130–134 10.1016/j.neulet.2007.10.044 18055112

[B71] Kennedy J. S. Buehner M. J. Rushton S. K. (2009). Adaptation to sensory-motor temporal misalignment: instrumental or perceptual learning? Q. J. Exp. Psychol. (Hove) 62, 453–69 10.1080/17470210801985235 18609410

[B72] King A. J. (2005). Multisensory integration: strategies for synchronization. Curr. Biol. 15, R339–R341 10.1016/j.cub.2005.04.022 15886092

[B73] King A. J. Palmer A. R. (1985). Integration of visual and auditory information in bimodal neurones in the guinea-pig superior colliculus. Exp. Brain Res. 60, 492–500 10.1007/BF00236934 4076371

[B74] Kinsler L. Frey A. Coppens A. (2000). Fundamentals of Acoustics. New York: Wiley

[B75] Kohler I. (1951). Formation and transformation of the perceptual world. Psychol. Issues 3, 1–173

[B76] Kopinska A. Harris L. R. (2004). Simultaneity constancy. Perception 33, 1049–1060 10.1068/p5169 15560507

[B77] Körding K. P. Beierholm U. Ma W. J. Quartz S. Tenenbaum J. B. Shams L. (2007). Causal inference in multisensory perception. PLoS ONE 2:e943 10.1371/journal.pone.0000943 17895984 PMC1978520

[B78] Lewald J. Guski R. (2004). Auditory-visual temporal integration as a function of distance: no compensation for sound-transmission time in human perception. Neurosci. Lett. 357, 119–122 10.1016/j.neulet.2003.12.045 15036589

[B79] Liverence B. M. Scholl B. J. (2012). Discrete events as units of perceived time. J. Exp. Psychol. Hum. Percept. Perform. 38, 549–554 10.1037/a0027228 22369229

[B80] Marinovic W. Arnold D. H. (2012). Separable temporal metrics for time perception and anticipatory actions. Proc. Biol. Sci. 279, 854–859 10.1098/rspb.2011.1598 21900323 PMC3259939

[B81] Matteson H. H. (1971). Effects of surround luminance on perceptual latency in the fovea. J. Opt. Soc. Am. 61, 1169–1172 10.1364/JOSA.61.001169 5121887

[B82] Meredith M. A. Nemitz J. W. Stein B. E. (1987). Determinants of multisensory integration in superior colliculus neurons. I. Temporal factors. J. Neurosci. 7, 3215–3229 3668625 10.1523/JNEUROSCI.07-10-03215.1987 PMC6569162

[B83] Michotte A. (1963). The Perception of Causality. Oxford: Basic Books

[B84] Moore J. Haggard P. (2008). Awareness of action: inference and prediction. Conscious. Cogn. 17, 136–144 10.1016/j.concog.2006.12.004 17306565

[B85] Moore J. W. Lagnado D. Deal D. C. Haggard P. (2009). Feelings of control: contingency determines experience of action. Cognition 110, 279–283 10.1016/j.cognition.2008.11.006 19110240

[B86] Moore J. W. Obhi S. S. (2012). Intentional binding and the sense of agency: a review. Conscious. Cogn. 21, 546–561 10.1016/j.concog.2012.10.005 22240158

[B87] Morrone M. C. Ross J. Burr D. (2005). Saccadic eye-movements cause relativistic compression of time as well as space. Nature 1–13 10.1038/nn1488 15965472

[B88] Navarra J. Hartcher-O’Brien J. Piazza E. Spence C. (2009). Adaptation to audiovisual synchrony modulates the speeded detection of sound. Proc. Natl. Acad. Sci. U.S.A. 106, 9123–9124 10.1073/pnas.0810486106 PMC2695059 19458252

[B89] Navarra J. Soto-Faraco S. Spence C. (2007). Adaptation to audiotactile asynchrony. Neurosci. Lett. 413, 72–76 10.1016/j.neulet.2006.11.027 17161530

[B90] New J. J. Scholl B. J. (2009). Subjective time dilation: spatially local, object-based, or a global visual experience? J. Vis. 9, 1–11 10.1167/9.2.4 19271914

[B91] Obhi S. S. Hall P. (2011). Sense of agency and intentional binding in joint action. Exp. Brain Res. 211, 655–662 10.1007/s00221-011-2675-2 21503647

[B92] O’Regan J. K. Noe A. (2001). A sensorimotor account of vision and visual consciousness. Behav. Brain Sci. 24, 939–973; discussion 973–1031. 10.1017/S0140525X01000115 12239892

[B93] Pacer M. Griffiths L. (2012). Elements of a rational framework for continuous-time causal induction. Proc. Annu. Conf. Cogn. Sci. Soc. 1, 833–838

[B94] Palmer S. E. (1999). Vision Science: Photons to Phenomenology. Cambridge, MA: Bradford Books/MIT Press

[B95] Pearl J. (2000). Causality: Models, Reasoning, and Inference, 2nd Edn New York: Cambridge University Press

[B96] Purpura K. Tranchina D. Kaplan E. Shapley R. M. (1990). Light adaptation in the primate retina: analysis of changes in gain and dynamics of monkey retinal ganglion cells. Vis. Neurosci. 4, 75–93 10.1017/S0952523800002789 2176096

[B97] Redding G. M. Rader S. D. Lucas D. R. (1992). Cognitive load and prism adaptation. J. Mot. Behav. 24, 238–246 10.1080/00222895.1992.9941618 12736129

[B98] Redding G. M. Rossetti Y. Wallace B. (2005). Applications of prism adaptation: a tutorial in theory and method. Neurosci. Biobehav. Rev. 29, 431–444 10.1016/j.neubiorev.2004.12.004 15820548

[B99] Redding G. M. Wallace B. (1993). Adaptive coordination and alignment of eye and hand. J. Mot. Behav. 25, 75–88 10.1080/00222895.1993.9941642 15064199

[B100] Redding G. M. Wallace B. (2002). Strategic calibration and spatial alignment: a model from prism adaptation. J. Mot. Behav. 34, 126–138 10.1080/00222890209601935 12057886

[B101] Scholl B. J. Tremoulet P. D. (2000). Perceptual causality and animacy. Trends Cogn. Sci. (Regul. Ed.) 4, 299–309 10.1016/S1364-6613(00)01506-0 10904254

[B102] Schulz L. Gopnik A. (2004). Causal learning across domains. Dev. Psychol. 40, 162–176 10.1037/0012-1649.40.2.162 14979758

[B103] Shams L. Beierholm U. R. (2010). Causal inference in perception. Trends Cogn. Sci. (Regul. Ed.) 14, 425–432 10.1016/j.tics.2010.07.001 20705502

[B104] Sloman S. A. (2005). Causal Models: How We Think About the World and its Alternatives. New York: Oxford University Press.

[B105] Spence C. (2010). “Prior entry: attention and temporal perception,” in Attention and Time, eds Nobre A. C. Coull J. T. (Oxford: Oxford University Press), 89–104

[B106] Spence C. Baddeley R. Zampini M. James R. Shore D. I. (2003). Multisensory temporal order judgments: when two locations are better than one. Percept. Psychophys. 65, 318–328 10.3758/BF03194803 12713247

[B107] Spence C. Squire S. (2003). Multisensory integration: maintaining the perception of synchrony. Curr. Biol. 13, R519–R521 10.1016/S0960-9822(03)00445-7 12842029

[B108] Stetson C. Cui X. Montague P. R. Eagleman D. M. (2006). Motor-sensory recalibration leads to an illusory reversal of action and sensation. Neuron 51, 651–659 10.1016/j.neuron.2006.08.006 16950162

[B109] Stevenson R. A. Fister J. K. Barnett Z. P. Nidiffer A. R. Wallace M. T. (2012). Interactions between the spatial and temporal stimulus factors that influence multisensory integration in human performance. Exp. Brain Res. 219, 121–137 10.1007/s00221-012-3072-1 22447249 PMC3526341

[B110] Stone J. V. Hunkin N. M. Porrill J. Wood R. Keeler V. Beanland M. (2001). When is now? Perception of simultaneity. Proc. Biol. Sci. 268, 31–38 10.1098/rspb.2000.1326 12123295 PMC1087597

[B111] Strother L. House K. A. Obhi S. S. (2010). Subjective agency and awareness of shared actions. Conscious. Cogn. 19, 12–20 10.1016/j.concog.2009.12.007 20122852

[B112] Sugano Y. Keetels M. Vroomen J. (2010). Adaptation to motor-visual and motor-auditory temporal lags transfer across modalities. Exp. Brain Res. 201, 393–399 10.1007/s00221-009-2047-3 19851760 PMC2832876

[B113] Sugita Y. Suzuki Y. (2003). Audiovisual perception: implicit estimation of sound-arrival time. Nature 421, 911 10.1038/421911a 12606990

[B114] Treisman M. (1963). Temporal discrimination and the indifference interval. Implications for a model of the “internal clock.” Psychol. Monogr. 77, 1–31 5877542 10.1037/h0093864

[B115] Tse P. U. Intriligator J. Rivest J. Cavanagh P. (2004). Attention and the subjective expansion of time. Percept. Psychophys. 66, 1171–1189 10.3758/BF03196844 15751474

[B116] van Eijk R. L. Kohlrausch A. Juola J. F. van de Par S. (2008). Audiovisual synchrony and temporal order judgments: effects of experimental method and stimulus type. Percept. Psychophys. 70, 955–968 10.3758/PP.70.6.955 18717383

[B117] van Wassenhove V. (2009). Minding time in an amodal representational space. Philos. Trans. R. Soc. Lond. B Biol. Sci. 364, 1815–1830 10.1098/rstb.2009.0023 19487185 PMC2685822

[B118] van Wassenhove V. Buonomano D. V. Shimojo S. Shams L. (2008). Distortions of subjective time perception within and across senses. PLoS ONE 3:e1437 10.1371/journal.pone.0001437 18197248 PMC2174530

[B119] van Wassenhove V. Grant K. W. Poeppel D. (2007). Temporal window of integration in auditory–visual speech perception. Neuropsychologia 45, 598–601 10.1016/j.neuropsychologia.2006.01.001 16530232

[B120] von Holst E. (1954). Relations between the central nervous system and the peripheral organs. Br. J. Anim. Behav. 2, 89–86 10.1016/S0950-5601(54)80044-X

[B121] Vroomen J. Keetels M. de Gelder B. Bertelson P. (2004). Recalibration of temporal order perception by exposure to audio-visual asynchrony. Brain Res. Cogn. Brain Res. 22, 32–35 10.1016/j.cogbrainres.2004.07.003 15561498

[B122] Waldmann M. R. Holyoak K. J. (1992). Predictive and diagnostic learning within causal models: asymmetries in cue competition. J. Exp. Psychol. Gen. 121, 222–236 10.1037/0096-3445.121.2.222 1534834

[B123] Wearden J. H. (2008). Slowing down an internal clock: implications for accounts of performance on four timing tasks. Q. J. Exp. Psychol. (Hove) 61, 263–274 10.1080/17470210701282576 17853194

[B124] Weiskrantz L. Elliott J. Darlington C. (1971). Preliminary observations on tickling oneself. Nature 230, 598–599 10.1038/230598a0 4928671

[B125] Welch R. B. (1978). Perceptual Modification. New York: Academic Press

[B126] Wencil E. B. Coslett H. B. Aguirre G. K. Chatterjee A. (2010). Carving the clock at its component joints: neural bases for interval timing. J. Neurophysiol. 104, 160–168 10.1152/jn.00029.2009 20457861 PMC2904232

[B127] Wenke D. Haggard P. (2009). How voluntary actions modulate time perception. Exp. Brain Res. 196, 311–318 10.1007/s00221-009-1848-8 19471909 PMC2700248

[B128] Wertheimer M. (1912). Experimentelle studien über das sehen von behwegung. Z. Psychol. 61, 161–265

[B129] Westheimer G. (1999). Discrimination of short time intervals by the human observer. Exp. Brain Res. 129, 121–126 10.1007/s002210050942 10550509

[B130] Westheimer G. McKee S. P. (1977). Perception of temporal order in adjacent visual stimuli. Vision Res. 17, 887–892 10.1016/0042-6989(77)90206-1 595393

[B131] Wittmann M. (1999). Time perception and temporal processing levels of the brain. Chronobiol. Int. 16, 17–32 10.3109/07420529908998709 10023573

[B132] Wohlschlager A. Engbert K. Haggard P. (2003). Intentionality as a constituting condition for the own self – and other selves. Conscious. Cogn. 12, 708–716 10.1016/S1053-8100(03)00083-7 14656512

[B133] Wolpert D. M. Ghahramani Z. (2000). Computational principles of movement neuroscience. Nat. Neurosci. 3(Suppl.), 1212–1217 10.1038/81497 11127840

[B134] Wozny D. R. Shams L. (2011). Recalibration of auditory space following milliseconds of cross-modal discrepancy. J. Neurosci. 31, 4607–4612 10.1523/JNEUROSCI.6079-10.2011 21430160 PMC3071751

[B135] Wundt W. (1874). Grundzuge der physiologischen Psychologie. Leipzig: W. Engelmann

[B136] Yarrow K. Johnson H. Haggard P. Rothwell J. C. (2004). Consistent chronostasis effects across saccade categories imply a subcortical efferent trigger. J. Cogn. Neurosci. 16, 839–847 10.1162/089892904970780 15200711 PMC1266050

[B137] Zampini M. Guest S. Shore D. I. Spence C. (2005). Audio-visual simultaneity judgments. Percept. Psychophys. 67, 531–544 10.3758/BF03193329 16119399

[B138] Zampini M. Shore D. I. Spence C. (2003). Audiovisual temporal order judgments. Exp. Brain Res. 152, 198–210 10.1007/s00221-003-1536-z 12879178

